# Novel roles for two-component regulatory systems in cytotoxicity and virulence-related properties in *Pseudomonas aeruginosa*

**DOI:** 10.3934/microbiol.2018.1.173

**Published:** 2018-03-08

**Authors:** Shaan L. Gellatly, Manjeet Bains, Elena B.M. Breidenstein, Janine Strehmel, Fany Reffuveille, Patrick K. Taylor, Amy T.Y. Yeung, Joerg Overhage, Robert E.W. Hancock

**Affiliations:** 1 Centre for Microbial Diseases and Immunity Research, Department of Microbiology & Immunology, University of British Columbia, Vancouver, BC, Canada; 2 Microbiology of Natural and Technical Interfaces Department, Institute of Functional Interfaces (IFG), Karlsruhe Institute of Technology (KIT), Karlsruhe, Germany

**Keywords:** *Pseudomonas aeruginosa*, two-component regulatory systems, cytotoxicity, motility, adherence, biofilm, FleSR, PilSR, WspR

## Abstract

The rapid adaptation of the opportunistic bacterial pathogen *Pseudomonas aeruginosa* to various growth modes and environmental conditions is controlled in part through diverse two-component regulatory systems. Some of these systems are well studied, but the majority are poorly characterized, even though it is likely that several of these systems contribute to virulence. Here, we screened all available strain PA14 mutants in 50 sensor kinases, 50 response regulators and 5 hybrid sensor/regulators, for contributions to cytotoxicity against cultured human bronchial epithelial cells, as assessed by the release of cytosolic lactate dehydrogenase. This enabled the identification of 8 response regulators and 3 sensor kinases that caused substantial decreases in cytotoxicity, and 5 response regulators and 8 sensor kinases that significantly increased cytotoxicity by 15–58% or more. These regulators were additionally involved in motility, adherence, type 3 secretion, production of cytotoxins, and the development of biofilms. Here we investigated in more detail the roles of FleSR, PilSR and WspR. Not all cognate pairs contributed to cytotoxicity (e.g. PhoPQ, PilSR) in the same way and some differences could be detected between the same mutants in PAO1 and PA14 strain backgrounds (e.g. FleSR, PhoPQ). This study highlights the potential importance of these regulators and their downstream targets on pathogenesis and demonstrates that cytotoxicity can be regulated by several systems and that their contributions are partly dependent on strain background.

## 1. Introduction

*Pseudomonas aeruginosa* is one of the most common pathogens found in health care settings. It is responsible for severe infections of compromised epithelial surfaces, including acute and chronic respiratory infections and severe burns. The vast majority of patients with a *Pseudomonas* lung infection have a serious underlying medical condition, such as neutropenia, obstructive pulmonary disease or cystic fibrosis [1],[2].

The ability of *P. aeruginosa* to inhabit and adapt to different environments is largely due to its very large repertoire of regulatory proteins, of which two-component regulatory systems (TCS) make up one of the largest families. These systems control practically all aspects of cellular function and multiple systems regulate various aspects of virulence, one facet of which is direct cytotoxicity to the host epithelium. *P. aeruginosa* secretes several enzymes that contribute to the death of host epithelial cells, including type 3 secreted ExoS, ExoT and ExoU, and type 2 secreted ExoA, and various proteases, lipases and phospholipases, as well as others [3],[4]. For example, strain PA14, used extensively here, is quite cytotoxic to epithelial cells largely due to a mutation in LadS [5] that leads to an upregulation in type 3 secreted cytotoxins, such as the acutely toxic ExoU, which accounts for much of this strain's cytotoxicity [6].

In the classical TCS, a membrane-bound sensor detects a ligand (signal), often externally and frequently uncharacterized, autophosphorylates at the cytoplasmic side of the membrane and then transfers the phosphate to a cytoplasmic response regulator, which in turn initiates a response, usually through the alteration of gene transcription. *P. aeruginosa* has 64 sensor kinases and 72 response regulators [7]. A few TCS have been characterized for their contribution to virulence or virulence-related phenotypes, particularly GacA-GacS, CbrA-CbrB, and PhoP-PhoQ, but relatively few have been shown to have direct contributions to cytotoxicity [7]–[9]. The vast majority of TCS remain uncharacterized and it is likely that many of these contribute some role to virulence. Here, we performed a survey study in which *P. aeruginosa* TCS mutants were screened for their altered ability to cause death to cultured lung epithelial cells, and selected regulators were subsequently examined in greater detail.

## 2. Materials and methods

### 2.1. Bacterial strains and growth

For the cytotoxicity screen, all available strain PA14 transposon mutants in sensor kinases or response regulators were obtained from the Harvard University mutant library [10]. We also examined *P. aeruginosa* strain PAO1 transposon mutants, including the *fleS* and *fleR* mutants from a mini-Tn5-*lux* library [11] and the *fliC* mutant from the University Washington library [12]. *P. aeruginosa* strain PAO1 mutants *phoQ*, *phoP*, and *phoQ* complemented with pucP::*phoQ^+^* (*phoQ^+^*) [9],[13],[14], and *fleR*, *fleS*, and *fleS* complemented with pUCP::*fleS^+^* (*fleS^+^*) were described previously [9],[13],[14]. All strains were grown in Luria-Bertani broth (LB) or Basal Medium 2 [BM2; comprising 7 mM (NH_4_)_2_SO_4_, 40 mM K_2_PO_4_, 22 mM KH_2_PO_4_, 20 mM glucose, 2 mM MgSO_4_, 40 μM FeSO_4_] at 37 °C overnight with agitation and containing applicable antibiotics: 15 μg/ml gentamicin for strain PA14 transposon mutants and the *phoP* and *phoQ* mutants in strain PAO1, 30 μg/ml tetracycline for other strain PAO1 transposon mutants, and 300 μg/ml carbenicillin for the *phoQ*^+^ complemented strain. For interaction assays, strains and mutants were grown to log phase [optical density at 600 nm, (OD_600_) of ∼0.5] in LB without antibiotics. Growth was measured automatically every 20 minutes by assessing turbidity at OD_620_ in a TECAN Spectrafluor Plus.

### 2.2. Complementation of the wspR mutant

For the complementation of *wspR* in PA14, the mini-Tn7 system was used [15],[16] whereby the mini-transposon Tn7 is inserted at a single *att*Tn7 site on the *P. aeruginosa* chromosome. A 1.4 kb DNA fragment containing the *wspR* gene was amplified with primers 3702F (5′-TGGCGGAGGTATTCGATTAG-3′) and 3702R (5′-TCGCGGTTTACTTCGACCAG-3′) and cloned into TOPO Zero-Blunt cloning vector (Invitrogen) and then into the pBBR1MCS vector. The endonuclease EcoRV was used to move the *wspR* gene fragment into a pUC18TminiTn7T-Tet plasmid to create pUC18TminiTn7T-Tet-*wspR^+^*. This vector was constructed by removing the tetracycline resistance gene from pFTc1 (AY712950) using the restriction enzyme *SacI* and ligating it into the *EcoRV* site of pUC18TminiTn7T (AY599226). The plasmid pUC18TminiTn7T-Tet-*wspR^+^* was then co-electroporated with the helper plasmid pTNS2 into electrocompetent PA14 *wspR* mutant cells. Positive clones containing the complementing gene were selected on LB plus 50 µg/ml of tetracycline. The insertions were checked by PCR using the primers P_TN7R_ and P*_glmS_*_-down_ as described previously [15],[16].

### 2.3. Cytotoxicity assays

The SV40-transformed, immortalized 16HBE14o-human bronchial epithelial cell line (HBE cells) was a gift from Dr. D. Gruenert (University of California, San Francisco, CA) [17]. These cells were grown as monolayers in minimal essential medium (MEM) with Earle's salts containing 10% fetal bovine serum (FBS) and 2 mM L-glutamine with phenol red (Gibco). HBE cells were seeded into 96-well tissue-culture treated plates and grown to confluence at 37 °C and 5% CO_2_ in minimal essential medium (MEM) containing 10% FBS and 2 mM L-glutamine with phenol red (Gibco). The monolayer was washed with MEM containing 1% FBS and 2 mM L-glutamine then rested for a minimum of 30 min in the same medium. Bacteria were grown to logarithmic phase in LB at 37 °C with agitation, numbers estimated using OD_600_ measurements, washed in PBS, and then resuspended in MEM containing 1% FBS and 2 mM L-glutamine. The HBE monolayer was infected at a multiplicity of infection (MOI) of 2 for 8–10 h (strain PA14) or at an MOI of 50 to 100 for 16–20 hr (strain PAO1), due to differences in the virulence of these strains. The HBE cells and bacteria were co-incubated at 37 °C and 5% CO_2_. After centrifugation to remove bacteria, media was collected and stored at 4 °C. Cytotoxicity was measured by the release of cytosolic lactate dehydrogenase (LDH) from epithelial cells into the media using a colorimetric kit (Roche Applied Sciences). The percentage cytotoxicity of the mutants was calculated from the ratio of cytotoxicity in the mutants to that of an uninfected control (0% lysis) and a 1% Triton X-100 lysis positive control (100% lysis).

Alterations in the expression of specific toxins were determined by quantitative real-time polymerase chain reaction (qRT-PCR) [18] using optimized primers, a list of which can be obtained from the authors.

### 2.4. Biofilm and motility assays

Static microtitre biofilm assays were performed as previously described [19],[20]. After overnight incubation at 37 °C without agitation in LB, media and non-adhered cells were discarded and the wells washed with deionized H_2_O. Surface-attached (biofilm) bacteria were stained with 0.1% crystal violet for 20 min, then washed again with deionized water. Crystal violet bound to the attached cells was dissolved with ethanol and the biofilm quantified by absorbance at 600 nm.

Flagellum-based swimming motility was analyzed by inoculating 1 μl of overnight culture into plates containing LB solidified with 0.25% agar and the in-agar swim zone diameter was measured after overnight incubation at 37 °C. Type IV pilus mediated twitching motility was assessed by inoculating 1 μl of an overnight culture through to the plastic surface of a thinly poured 1% agar LB plate and, after overnight incubation at 37 °C, measuring the diameter of the twitch zone that formed at the interface between the plastic surface and the agar. Swarming motility was carried out on BM2-glucose plates containing 0.5% agar and, in place of (NH_4_)_2_SO_4_, 0.1% casamino acids for strain PA14 and 0.5% casamino acids for strain PAO1. One μl of overnight culture was inoculated onto the surface of swarm plates that were then incubated at 37 °C overnight.

### 2.5. Adherence to epithelial cells

HBE cells and bacteria for infection were prepared as described above for the cytotoxicity assay. Bacteria were added to the HBE monolayers at MOI 50 and co-incubated at 37 °C and 5% CO_2_. After 1 hour, the media was removed and the monolayer washed thrice with PBS to remove any un-adhered bacteria [21]. The monolayer was lysed with 2% triton X-100 in PBS and removed and immediately diluted for plating onto LB agar as the adhered fraction. The inoculum was also plated to estimate total bacteria. Adherence was assessed as a percentage of adhered compared to total bacteria.

## 3. Results

### 3.1. Cytotoxicity profiles of PA14 two-component system mutants

*P. aeruginosa* causes both acute opportunistic lung infections as well as chronic lung infections in humans. In such infections, there is often considerable damage to the epithelium of the lung mediated by a variety of toxins and secreted enzymes [1],[2]. Two-component regulatory systems (TCS) provide *Pseudomonas* with the ability to rapidly adjust to changing conditions in the host and other environments, and in this regard likely control many aspects of *P. aeruginosa* cellular life and virulence. One aspect of *P. aeruginosa* virulence is cytotoxicity towards host cells, including epithelial cells, through injected or secreted toxins. To measure this cytotoxic effect, human bronchial epithelial (HBE) cells were grown to confluence in culture and infected with *P. aeruginosa* strain PA14 at a MOI of 2 for 8 hours. At this time, necrosis of the HBE cells, as assessed by microscopy, was starting to become apparent as indicated by the emergence of lesions in the monolayer (data not shown). Cytotoxicity was quantitatively assessed by measuring the release of cytosolic lactate dehydrogenase (LDH) using a colorimetric assay, background subtracted for the release by untreated HBEs, and compared to a Triton X-100 lysis control. In all, 50 sensor kinases, 50 response regulators, 5 hybrid sensor/regulators, and 4 other TCS regulator mutants from the Harvard University strain PA14 transposon library were screened for cytotoxicity to HBE cells (Table S1). The ability to grow under co-culturing conditions was assessed spectrophotometrically at the end of the incubation time (data not shown); only one mutant (PA2571) was found to have a growth defect under these conditions and was therefore excluded.

Mutants in 13 regulators (11 response regulators and 2 other regulators), 10 sensor kinases and one sensor/regulator hybrid demonstrated substantial (>15%) alterations in their ability to cause lysis of epithelial cells compared to WT (Table 1). Of those causing increased cytotoxicity by 17–58%, eight were sensor kinases (including one hybrid) and 5 were response regulators, but intriguingly there were only 2 well studied cognate pairs, GacAS and CbrAB. Conversely, the TCS regulators that suppressed cytotoxicity were less balanced with 8 response regulators and only 3 sensor kinases, including one cognate triad, FleQRS. Thus there were many instances where only a single TCS component could be shown to have an effect on cytotoxicity (e.g. PhoQ but not PhoP; PA3044 but not PA3045; PilR but not PilS), which indicated that they were cross-talking with other TCS components. Other TCS components were either orphans (no adjacent senor kinase or response regulator), perhaps also acting through cross talk to another TCS component, or in a few instances the mutant in the second component was not available. Interestingly there was one instance (WspR-WspE) where the sensor kinase and response regulator mutants had opposite effects, although for the Wsp system there are substantial doubts as to whether these represent a cognate pair. Intriguingly there were only 2 un-annotated TCS components identified in the collection to have toxicity phenotypes, and both led to increased cytotoxicity.

Overall, we demonstrated an effect on cytotoxicity of TCS components with known involvement in type IV pili (*pilG*, *pilH*, *pilR*) and flagella-mediated motility (*fleQ*, *fleS*, *fleR*), virulence (*gacS*, *gacA*, *phoQ*, *algR*, *cbrA*, *mifS*, *algR*), cyclic-di-GMP signaling (*wspR*, *rocA1*, *fleQ*, *wspE*), and metabolism (*cbrAB*, *dctB*, *ntrBC*, *fpvR*). In particular, the well-known virulence regulator AlgR of the AlgR-FimS TCS, showed a 67% decrease in cytotoxicity. This role for AlgR joins a wide range of other functions in several aspects of *P. aeruginosa* virulence, including regulation of T3SS [22],[23], likely explaining its cytotoxicity phenotype, alginate and LPS biosynthesis, biofilm formation, hydrogen cyanide production [24],[25], and twitching and swarming motilities [7],[19], as well as regulation of virulence in murine models [26]. A mutant in the sensor kinase-encoding gene *dctB* (PA5165) showed only 13% of the cytotoxicity of WT (Table 1). The DctB-DctD two-component system has been identified in *P. aeruginosa* as having a role in C4-dicarboxylate transport [27]–[29], however intriguingly the *dctD* mutant had no cytotoxicity defect. Conversely, the probable sensor kinase PA2882 (139% cytotoxicity of WT) has weak homology to the binding domain found in sensors of C4-dicarboxylates.

**Table 1.** Effect of strain PA14 mutations in two-component regulatory system kinases and response regulators on cytotoxicity towards human bronchial epithelial cells. Shown are those mutants that demonstrated >15% change in cytotoxicity compared to WT. In general 3 or more experiments were performed assessing cytotoxicity (measured as LDH release after 8 hours of infection); results are expressed as the mean ± standard error expressed as a percentage of cytotoxicity caused by WT.

PA14 locus ID	PAO1 homolog	Gene name	Description	Mean % of WT ± standard error
Mutants with increased cytotoxicity				
PA14_07840	PA0601	*agtR*	Two-component response regulator; contains a CheY-like receiver domain; amine uptake	119.9 ± 4.2
PA14_52260	PA0928	*gacS*	Sensor/response regulator hybrid; multi-host virulence through regulation of small regulatory RNAs RsmZ and RsmY	134.3 ± 4.7
PA14_49170	PA1180	*phoQ*	Two-component sensor kinase PhoQ	121.1 ± 5.7^‡^
PA14_45590	PA1458		Probable two-component sensor kinase; putative homolog of *E. coli* chemotaxis regulator CheA	144.3 ± 6.3
PA14_33780	PA2388	*fpvR*	Probable Fe^2+^-dicitrate sensor kinase	121.6 ± 7.9
PA14_30650	PA2586	*gacA*	Response regulator; multi-host virulence through regulation of small regulatory RNAs RsmZ and RsmY	144.7 ± 15.8
PA14_26810	PA2882		Probable two-component sensor kinase; has binding domain homologous to that found in sensors of C4-dicarboxylates	139.3 ± 15.7
PA14_16470	PA3704	*wspE*	CheA-type sensor kinase; c-di-GMP regulation	134.5 ± 10.4
PA14_62530	PA4725	*cbrA*	Two-component sensor kinase; required for carbon-nitrogen balance and control of catabolite repression	158.8 ± 9.1
PA14_62540	PA4726	*cbrB*	Two-component response regulator; required for carbon-nitrogen balance and control of catabolite repression	140.8 ± 9.7
PA14_67680	PA5125	*ntrC*	Two-component response regulator; regulates use of carbon and nitrogen	122.6 ± 4.1
PA14_67670	PA5124	*ntrB*	Two-component sensor kinase; regulates use of carbon and nitrogen	117.1 ± 5.8
PA14_70790	PA5364		Probable two-component response regulator	130.4 ± 9.1
PA14_72740	PA5512	*mifS*	Two-component sensor kinase; regulates biofilm development	119.6 ± 13.2
Mutants with decreased cytotoxicity				
PA14_05320	PA0408	*pilG*	Type IV pilus response regulator; required for pilus extension and twitching motility	28.8 ± 4.4
PA14_05330	PA0409	*pilH*	Type IV pilus response regulator; required for pilus retraction	25.8 ± 1.5
PA14_50220	PA1097	*fleQ*	Flagella major transcriptional regulator; cyclic-di-GMP responsive; potential FleSR modulator	77.6 ± 12.7
PA14_50200	PA1098	*fleS*	Two-component sensor kinase; required for hook and basal body protein biosynthesis for flagellum assembly	20.6 ± 6.7^‡^
PA14_50180	PA1099	*fleR*	Two-component response regulator; required for hook and basal body protein biosynthesis for flagellum assembly	12.3 ± 0.8^‡^
PA14_16500	PA3702	*wspR*	Two-component response regulator with GGDEF domain; c-di-GMP regulation	16.8 ± 3.7
PA14_12780	PA3948	*rocA*	Two-component response regulator; cyclic-di-GMP regulation	53.2 ± 0.9
PA14_60260	PA4547	*pilR*	Two-component response regulator; required for pilus expression and therefore for type IV pilus biosynthesis	14.6 ± 4.5
PA14_68230	PA5165	*dctB*	Two-component sensor kinase; regulates a C4-dicarboxylate transport system with DctD	14.9 ± 3.5
PA14_69470	PA5261	*algR*	Alginate biosynthesis regulatory protein	33.2 ± 11.7

^‡^ Mutants are listed in order of the PAO1 homolog.

To determine the possible mechanisms underlying altered cytotoxicity, we assessed the regulation by selected TCS components of several known virulence factors (elastase, type 3 secretion, lipase, exotoxin A), each of which are known to exert independent cytotoxic effects on host cells (Table 2). Furthermore, we assessed whether there was a defect in adherence to host epithelial cells that might impact on cytotoxicity (Table 3). Overall these studies demonstrated the altered expression of exotoxin A (*fleS*), type 3 secretion (*fleQ*, *fleR*, and *fleS*) and elastase genes (*fleQ*, *fleR*, and *fleS*), as well as defects in adherence (*fleS*, *fliC*, *pilB*, *wspR*). We concluded that generally speaking there was no single mechanism operating in the mutants, and that selected TCS regulators were able to influence multiple mechanisms of cytotoxicity.

### 3.2. A regulator of flagellum biosynthesis affected cytotoxicity, biofilm formation and motility

**Figure 1.**
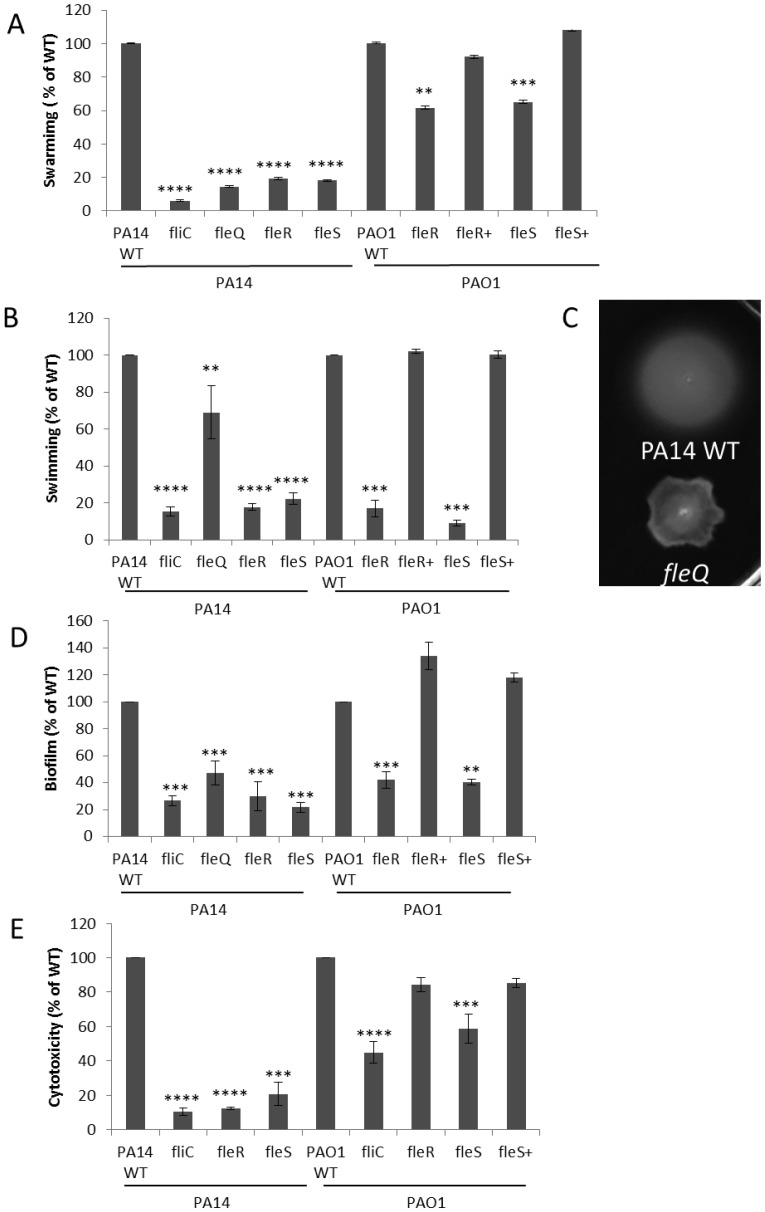
Flagella regulatory mutants demonstrated deficiencies in swarming (A) and swimming (B, C) motility, biofilm formation (D) and cytotoxicity (E). Data shown is with strains PA14 and PAO1. Swarming was performed on BM2-glucose containing casamino acids and 0.5% agar; as the two strains exhibit different swarming behaviours the data is shown as a representative photo for PA14 (dendritic movement) and a bar graph for PAO1 (circular movement). Swimming was measured as the ability of bacteria to move through LB-containing 0.25% agar. A non-flagellated *fliC* mutant was used as a control (B). A *fleQ* mutant demonstrated aberrant swimming motility (C). Intriguingly, mutations in *fleR* and *fleS* had a more pronounced effect on swarming (panels A and B) and cytotoxicity (panel E) in PA14 than in PAO1. Student's t-test, ***p < 0.001.

A decrease in cytotoxicity was demonstrated for mutants in the flagella-regulating TCS, FleS-FleR (21% and 12% of WT respectively) (Table 1). The FleS-FleR system controls the expression of genes encoding the basal body, hook and filament proteins in flagellum biogenesis. Neither *fleS* nor *fleR* mutants produce flagella and both have major swimming [30] and swarming (PA14 only; Figure 1A) deficiencies, as confirmed here by comparison to the control non-flagellated mutant, *fliC* (Figure 1B).

The bacterial flagellum is exported via a system that is ancestrally related to the T3SS [31],[32] which in *P. aeruginosa* is responsible for secreting four known virulence effectors, ExoT, ExoY, ExoS, and ExoU [1], the latter of which is strongly cytotoxic and is expressed in strain PA14. To investigate potential overlaps between flagella and T3SS, qPCR was performed on the *fleQ*, *fleR*, and *fleS* mutants, revealing 1.9-fold to 3.5-fold decreases in the expression of *exsA*, the master T3SS regulator (Table 2). Therefore the *fleQRS* system likely plays a role in regulating the T3SS, which in turn has an impact on cytotoxicity.

In addition to their overlapping effects on the T3SS, flagella are involved in adherence and motility. Flagella as well as pili are required for biofilm formation [20] and swarming motility [33]. Similar to the trends shown for swimming and cytotoxicity, *fleR* and *fleS* mutants demonstrated approximately 4-fold reductions in biofilm formation (Figure 1D). Both mutants also demonstrated substantial deficiencies in swarming (Figure 1A), consistent with the known role that FleRS plays in controlling flagella expression [34]. Given the known role of flagella in adherence to surfaces, we could demonstrate that the *fleS* mutant had a major defect in adherence to HBE cells (Table 3); surprisingly, the *fleR* mutant demonstrated only a marginal defect while the upstream *fleQ* mutant had a strong but more modest decrease in adherence (Table 3) despite its major effect on adherence to mucin [35].

Mutants in both *fleR* and *fleS* and their complemented counterparts were available in strain PAO1 and have previously been published by this lab [14]. Strain PAO1 is less virulent and far less cytotoxic than strain PA14, and correspondingly the disruption of *fleS* in strain PA14 resulted in a more substantial reduction in cytotoxicity compared to WT (20.6%) than in PAO1 [58%; although this could be complemented to 85% of WT (Figure 1E)]. In contrast to its effect in strain PA14, the *fleR* PAO1 mutant showed very little change in cytotoxicity. Sequence alignment demonstrated 100% identity (comparing strains PA14 and PAO1) in the amino acid sequences for FleR, and a single threonine to proline substitution at the N-terminus in FleS. As with strain PA14, both *fleR* and *fleS* PAO1 mutants demonstrated downregulation of the T3SS (*exsA*; Table 2) and like PA14, only the *fleS* mutant showed a substantial adherence deficiency. These data further supported the conclusions that one basis for the reduced virulence observed in PAO1 might be altered regulation in the two different strain backgrounds. Consistent with the concept of differential regulation in these two strains, a *phoQ* mutant in strain PAO1 was substantially less cytotoxic [9] but the same mutant showed increased cytotoxicity in strain PA14 (Table 1). Similarly a *dctB* mutant showed no deficiency in strain PAO1 (data not shown) but a substantial decrease in cytotoxicity in strain PA14 (Table 1).

**Table 2.** Regulation of selected known virulence genes in mutants compared to the WT. The effects of various mutations on expression of *lipC* (lipase C), *toxA* (exotoxin A), *exsA* (master regulator of T3SS), compared to the control (*pilS*) were detected by qRT-PCR. Shown is the mean ± standard error. Student's t-test where *p < 0.05, **p < 0.01, ***p < 0.001, and ****p < 0.0001.

Mutant	Fold change compared to WT			
PA14	*lipC*	*toxA*	*exsA*	*lasB*
*fleQ*	−1.54 ± 0.17	1.29 ± 0.12	−1.89 ± 0.10****	−1.89 ± 0.24***
*fleR*	1.10 ± 0.03	1.45 ± 0.21	−2.16 ± 0.09***	−1.33 ± 0.15***
*fleS*	−1.57 ± 0.10	−3.36 ± 0.28***	−2.74 ± 0.48****	−2.25 ± 0.23***
*pilS*	−1.03 ± 0.05	1.55 ± 0.39	−1.42 ± 0.22	−1.7 ± 0.36
PAO1				
*fleR*	−1.13 ± 0.28	−1.45 ± 0.02	−2.7 ± 0.89**	−2.42 ± 1.64
*fleS*	1.15 ± 0.27	−1.45 ± 0.47	−3.49 ± 0.37***	−2.84 ± 0.66**

FleQ is upstream of FleRS and has been proposed to act in a regulatory cascade with FleR in controlling motility and adhesion in *P. aeruginosa*. The *fleQ* mutant demonstrated a more modest reduction in cytotoxicity compared to either *fleR* or *fleS* (22% vs. 79–88%). Intriguingly although FleQ has been termed a master flagella regulator [35], mutation of *fleQ* had only a modest effect on swimming, consistent with its more modest decrease in cytotoxicity compared to the *fleR* and *fleS* mutants. Indeed the swimming phenotype observed was unusual (Figure 1C), displaying an uneven spread through the medium. Conversely, a mutant in the probable sensor PA1458 [33], which has been suggested to be a homolog of *E. coli* chemotaxis regulator CheA and is expressed in a FleQ dependent manner [34], demonstrated considerably higher cytotoxicity (144% of WT). This transposon mutant also displayed deficiencies in swimming and swarming motilities, but not in biofilm formation [19].

### 3.3. Mutants in type IV pili demonstrated decreased cytotoxicity

**Figure 2.**
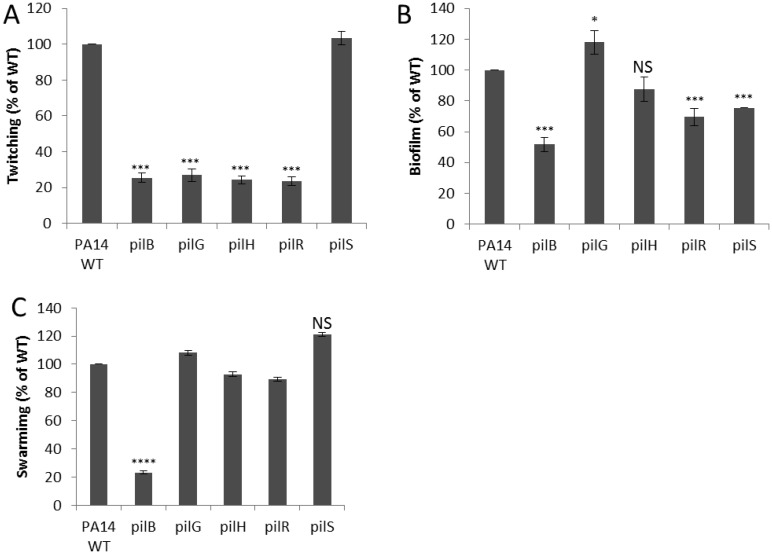
Effect of mutants in regulators of type IV pili on twitching, biofilm formation and swarming. Various regulators showed alterations in some but not all of these pilus-related phenotypes. (A) Twitching motility along the agar-plastic interface of LB plates containing 1% agar was reduced in *pilG*, *pilH* and *pilR* regulator mutants but not in *pilS*. The non-piliated *pilB* was used as a control. (B) Pilus-related regulatory mutants demonstrated alterations in their abilities to produce static biofilm. Overnight cultures were diluted into LB and grown for 24 hours in polystyrene 96-well plates. The cultures were then removed, the wells washed, and the remaining biofilm stained with crystal violet. The amount of crystal violet bound corresponded directly to the amount of biofilm formed. (C) Swarming motility across the surface of semi-solid 0.5% agar. Only the non-piliated *pilB* mutant demonstrated a reduction in swarming. Student's t-test, mean average with standard error. *p < 0.05, **p < 0.01, NS not significant.

The PilS-PilR two-component system controls the transcription of *pilA* which encodes the type IV pilin subunit [36]. Bacterial pili are responsible for adherence to surfaces, twitching motility and biofilm formation. A *pilR* response regulator mutant demonstrated only 15% of the cytotoxicity of WT, while a mutant in the sensor kinase *pilS* showed no differences. Such a difference could indicate either that the sensor kinase PilS negatively regulated PilR, or that PilR cross-talked with a second sensor kinase. The *pilGHIJKL-chpABCDE* chemosensory gene cluster also contributes to the biogenesis of type IV pili, as well as to the direction and pattern of twitching motility, which are known to be essential for non-opsonic phagocytosis [37], initial attachment to inert surfaces, biofilm formation, and pathogenesis in many models [20],[38]–[41]. The type IV pilus regulators, *pilG* and *pilH* demonstrated respectively 29% and 26% of the cytotoxicity of WT. Consistent with these cytotoxicity data, the *pilG, pilH*, and *pilR* mutants, but not the *pilS* mutant, were deficient in twitching motility (Figure 2A). In contrast, regarding biofilm formation there was a different trend (Figure 2B) since both *pilR* and *pilS* mutants demonstrated reductions in biofilm formation, while the *pilG* mutant demonstrated a small increase in biofilm formation. Conversely swarming motility, which requires type IV pili [19],[33], was not affected by any of these regulators, but was affected in the non-piliated control mutant, *pilB* (Figure 2C). None of the pilus regulators tested had a statistically significant defect in adherence to HBE cells, in contrast to the *pilB* mutant that was very deficient (Table 3). This indicates that the observed cytotoxicity defects in these regulator mutants likely related to their differential ability to interact with epithelial cells rather than the presence or absence of pili per se.

### 3.4. Cytotoxicity was affected by the Wsp chemosensory regulators that affect c-di-GMP

The Wsp chemosensory-type two component regulator is involved in cyclic-di-GMP (c-di-GMP) signaling networks. Instead of the DNA-binding domain of classic two-component regulators, WspR contains an enzymatic GGDEF diguanylate cyclase domain [40],[42]. A mutant in the *wspR* response regulator displayed only 16.8% of the cytotoxicity of the WT, while a mutant in its cognate CheA-type sensor kinase WspE demonstrated a strongly increased cytotoxicity to 134% of WT (Table 1; N.B. another CheA homolog PA1458 also had a strong increase in cytotoxicity as described above). Analysis of known toxin secretion pathways by qPCR demonstrated a 2-fold increase in the type 3 secretion gene *pscD* for the *wspE* mutant which may explain its increased cytotoxic ability.

**Figure 3.**
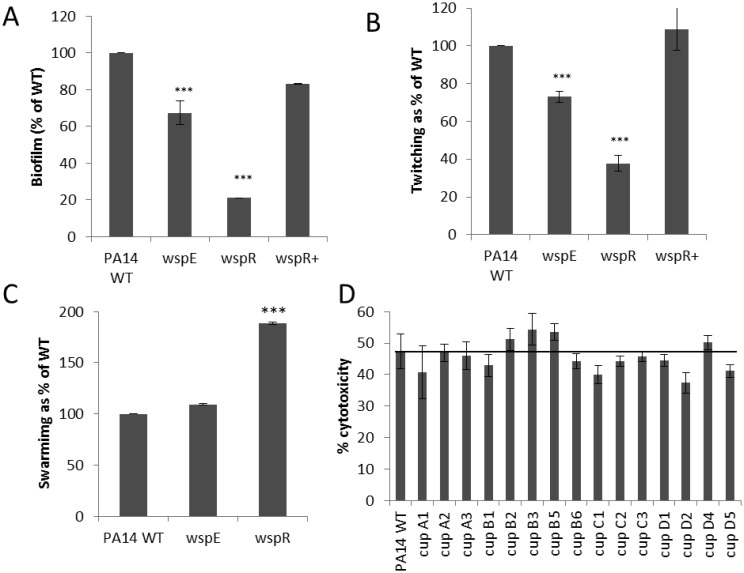
The effect of the Wsp chemosensory system on biofilm formation (A) and twitching motility (B). A lesser (stimulatory) effect was observed for swarming motility (C). Available Cup fimbriae mutants did not show any significant abnormalities in their cytotoxic abilities towards cultured HBE cells (D). Student's t-test, ***p < 0.001.

C-di-GMP has been proposed to affect functions related to the switch between the motile, single cell state and the surface-associated multicellular biofilm state [43]. The levels of c-di-GMP are regulated by GGDEF domain-containing diguanylate cyclases (e.g. WspR) and EAL-domain containing c-di-GMP phosphodiesterases [40],[42]. Increased levels of c-di-GMP have been associated with increased matrix production and biofilm formation while low levels of c-di-GMP downregulate matrix production and promote a planktonic lifestyle [40]. For this reason, the ability of the Wsp mutants to form biofilms and mediate various motilities was tested. The *wspR* mutant was nearly completely impaired in biofilm formation, while *wspE* reduced biofilm formation to two thirds of WT (Figure 3A). Similarly a *wspR* mutant was severely impaired in twitching, while *wspE* led to only a modest defect in twitching (Figure 3B). Both the twitching and biofilm deficiencies of *wspR* could be restored to WT levels through complementation. The opposite effects of *wspR* and *wspE* mutants on cytotoxicity, but similar effects on twitching and biofilm formation indicated that these properties are differentially regulated. There was no effect on swarming for *wspE*, but *wspR* demonstrated a substantial increase (188% of WT). Swimming was unaffected (data not shown) and only the *wspR* mutant was deficient in adherence to HBE cells (Table 3).

**Table 3.** Adherence of TCS mutants to HBE cells after 1 hour. Student's t-test where *p < 0.05, **p < 0.01, ***p < 0.001, and ****p < 0.0001 compared to wildtype.

Strain	% Adhered ± SEM
PA14 WT	7.6 ± 1.6
*algR*	0.3 ± 0.2*
*cbrA*	2.0 ± 0.4*
*cbrB*	2.8 ± 0.6
*dctB*	0.3 ± 0.2*
*fleQ*	1.1 ± 0.4*
*fleR*	2.5 ± 1.0*
*fleS*	0.2 ± 0.1****
*fliC*	0.5 ± 0.4*
*fpvR*	1.7 ± 0.4*
*gacA*	4.1 ± 1.1
*gacS*	6.6 ± 1.7
*mifS*	2.5 ± 1.2
*ntrB*	1.6 ± 1.3*
*ntrC*	1.1 ± 0.1*
*phoQ*	2.6 ± 0.4
*pilB*	1.8 ± 0.5*
*pilG*	6.7 ± 1.4
*pilH*	2.7 ± 0.7*
*pilR*	0.3 ± 0.1*
*pilS*	8.1 ± 1.2
*rocA1*	1.7 ± 0.6*
*wspE*	7.6 ± 2.7
*wspR*	0.5 ± 0.2**
PA1458	4.4 ± 0.4
PA2282	2.7 ± 0.7
PA5364	2.9 ± 1.6
PAO1 WT	8.4 ± 1.4
*fleS*	0.5 ± 0.2*
*flesR*	10.0 ± 1.2

Signaling via the Wsp chemosensory system (WspABCDEF) results in the activation of the WspR regulator causing the upregulation of adhesion factors, including the *cupA* gene cluster (one of five Cup fimbrial clusters in *P. aeruginosa*) [42],[44] that was previously demonstrated to have a role in biofilm formation [39]. However individual mutants in Cup fimbria genes showed no major alterations in cytotoxicity (Figure 3D), indicating that cytotoxicity was related to other aspects of c-di-GMP regulation. It is possible that the twitching, biofilm and adherence deficiencies of *wspR* indicated that the Wsp chemosensory system plays a role in regulating type IV pili.

In addition, a *rocA1* (also called *sadA*) response regulator mutant also showed modestly decreased cytotoxicity (53% of WT), although mutants in its neighboring genes *rocR* (sensor kinase) *rocS* (encoding an EAL domain) showed no effects, indicating that its effect on cytotoxicity might be independent of c-di-GMP signalling. Alternatively it is possible that other c-di-GMP producing proteins can be regulated directly or indirectly by *rocA1* and thus c-di-GMP could still be involved.

## 4. Conclusions

*P. aeruginosa* is able to cause severe infections at any compromised epithelial site. The pathogenesis and the outcome of such an infection are influenced by the host immune response and the virulence factors of the infectious organism. These virulence factors are controlled in part by bacterial TCSs that can rapidly adjust bacterial physiology to changing medium conditions. Here we performed an initial survey study to demonstrate TCS regulation of cytotoxicity was quite broad. Thus, TCS mutants of *P. aeruginosa* strain PA14 were co-cultured with lung epithelial cells with the purpose of uncovering which TCSs were involved in the necrotic killing of these epithelial cells. In particular, the genes influencing cytotoxicity in PA14 seemed to be grouped into several themes: control of carbon and nitrogen utilization (*dctB*, *cbrAB*, *ntrBC*), motility and adherence (*pilG*, *pilH*, *pilR*, *fleQ*, *fleR*, *fleS*), and c-di-GMP signaling (*wspR*, *wspE*, *rocA1*).

Several of the TCS mutants that demonstrated reduced cytotoxicity were involved in adherence or motility either directly, as for the flagella and pilus regulators, or indirectly, such as the regulators of c-di-GMP, a bacterial secondary messenger known to reciprocally regulate adherence and motility [43]. The observation that adherence and motility are involved in various aspects of virulence such as cytotoxicity is well known, however the data here highlight the importance of strain variations when studying the contribution of TCSs to cytotoxicity. This cytotoxicity screen was performed in strain PA14 due to the availability of a reliable and quite comprehensive PA14 transposon mutant library [10]. A few genes, where mutants were available, were also analyzed in the considerably less toxic strain PAO1. Genes directly involved in the adherence of PAO1 or PA14 (e.g. flagellum and pilus synthesis/function) largely showed decreased cytotoxicity. In contrast, the comparison of PA14 and PAO1 mutants in *phoQ* (Table 1, Table S1) and *fleR* (Figure 1E) demonstrated a large variability in the control of cytotoxicity by these regulatory systems between the two strain backgrounds. This discrepancy might be in a large part a result of differences in the T3SS, due to the high cytotoxic capability of ExoU that is present only in strain PA14 [45]. Our own studies have shown that cytotoxicity in the PAO1 background is only partly due to the T3SS, and likely works through other toxic enzymes such as lipases and proteases, as indicated by the large reduction in the cytotoxicity of mutants in these exoenzymes in PAO1 [21]. Nonetheless, few TCSs were analyzed in PAO1 due to the limited availability of reliable mutants in that strain at the time the screen was performed (there were numerous anecdotal reports [46],[47] and our own lab's experience that mutants of this library had become cross contaminated). It would thus be interesting to expand the screen into PAO1, as reliable mutants become available, to see which other TCSs show substantial differences in this strain background.

A key finding here was that not all predicted sensor/regulator pairs demonstrated equivalent changes in cytotoxicity. For example, mutants of *pilR*, *dctB* and *phoQ* demonstrated a change in cytotoxicity to HBE cells compared to the WT, yet their cognate partners, *pilS*, *dctD* and *phoP*, did not. TCSs can have very substantial regulons, and different systems might have some overlap with others [7]–[9]. It is conceivable that some TCSs might play more of a global regulatory role, whereby, for example, a sensor controls a large regulon by phosphorylating its cognate response regulator as well as other response regulators, or phosphorylates an adapter protein which relays signals between regulatory networks. Certainly, cytotoxicity appears to be regulated by many systems (Table 1). Indeed we previously demonstrated that CbrA regulated susceptibility to 3 antibiotics and persistence and virulence in animal models, independently of its adjacent TCS partner CbrB, although these TCS partners jointly regulated several other properties including cytotoxicity, biofilm formation, swarming motility and catabolite repression [8]. Intriguingly although chronic infections are associated with high biofilm capacity and low cytotoxicity while acute infections are the opposite, we identified TCS regulators that regulated both elements in the same direction indicating that these processes can be coordinately regulated. Comparisons of RNA-Seq performed on different mutants known to regulate cytotoxicity could help to describe the disparate signaling networks that in turn might enable identification of potential therapeutic targets.

The use of transposon libraries carries with it the strong potential for phenotypes to be incorrectly assigned to a particular gene due to a polar effect of the transposon on expression of a downstream gene or genes within an operon. As TCS sensors and regulators are quite often transcribed together in an operon, complementation studies of *fleS*, *fleR*, and *wspR* were performed in part to address the possibility of polar effects in these systems. Despite this, some polar effects can be difficult to identify. For example, in *P. aeruginosa* the disruption of *phoP* has been shown to exert a polar effect on downstream *phoQ* due to an overlap in the genes, but only the targeted mutation of *phoQ* results in a major phenotypic difference [13],[48]. As not all of the TCSs studied here were chosen for further analysis, it is possible that in a few cases polar effects on downstream genes led to the observed cytotoxicity phenotype.

Although *in vitro* studies are often used to predict the effect of a mutation or substance in a host system, increased toxicity towards host cells does not always equate with increased virulence *in vivo.* For example, deletion of *cbrA* demonstrated an increase in *in vitro* cytotoxicity but a decrease in pathogenesis in a murine acute lung infection model [49]. Cell cultures do not accurately reflect the complexity of the host environment with its multitude of different cell types and secreted mediators. In addition, particularly with an opportunistic pathogen such as *P. aeruginosa*, the pathogenesis of any given mutant might be markedly altered in one *in vivo* infection model but not in another (e.g. acute lung vs. chronic lung). Due to this, it is possible that many TCS components screened in this study that demonstrated no observed change in cytotoxicity may indeed contribute to virulence in an *in vivo* model. Notwithstanding, it is far too costly and time consuming for broad *in vivo* screens to be practical, meaning that for now, molecular methods must be pursued.

Overall the regulation of cytotoxicity, motility and biofilm formation is very complex and highly interlinked. Two component regulators play a special role, likely related to their special ability to rapidly transduce environmental signals into altered bacterial lifestyles.

## Figures and Tables

**Table S1.** Effect of strain PA14 mutations in two-component regulatory system kinases and response regulators on cytotoxicity towards human bronchial epithelial cells. In general 3 or more experiments were performed assessing cytotoxicity (measured as LDH release after 8 hours of infection); results are expressed as the mean ± standard error expressed as a percentage of cytotoxicity caused by WT.

PA14 locus ID	PAO1 homolog	Gene name	Description	Mean % of WT ± standard error
Mutants with increased cytotoxicity				
PA14_07840	PA0601	*agtR*	Two-component response regulator; contains a CheY-like receiver domain; amine uptake	119.9 ± 4.2
PA14_52260	PA0928	*gacS*	Sensor/response regulator hybrid; multi-host virulence through regulation of small regulatory RNAs RsmZ and RsmY	134.3 ± 4.7
PA14_49170	PA1180	*phoQ*	Two-component sensor kinase PhoQ	121.1 ± 5.7^‡^
PA14_45590	PA1458		Probable two-component sensor kinase; putative homolog of *E. coli* chemotaxis regulator CheA	144.3 ± 6.3
PA14_33780	PA2388	*fpvR*	Probable Fe^2+^-dicitrate sensor kinase	121.6 ± 7.9
PA14_30650	PA2586	*gacA*	Response regulator; multi-host virulence through regulation of small regulatory RNAs RsmZ and RsmY	144.7 ± 15.8
PA14_26810	PA2882		Probable two-component sensor kinase; has binding domain homologous to that found in sensors of C4-dicarboxylates	139.3 ± 15.7
PA14_16470	PA3704	*wspE*	CheA-type sensor kinase; c-di-GMP regulation	134.5 ± 10.4
PA14_62530	PA4725	*cbrA*	Two-component sensor kinase; required for carbon-nitrogen balance and control of catabolite repression	158.8 ± 9.1
PA14_62540	PA4726	*cbrB*	Two-component response regulator; required for carbon-nitrogen balance and control of catabolite repression	140.8 ± 9.7
PA14_67680	PA5125	*ntrC*	Two-component response regulator; regulates use of carbon and nitrogen	122.6 ± 4.1
PA14_67670	PA5124	*ntrB*	Two-component sensor kinase; regulates use of carbon and nitrogen	117.1 ± 5.8
PA14_70790	PA5364		Probable two-component response regulator	130.4 ± 9.1
PA14_72740	PA5512	*mifS*	Two-component sensor kinase; regulates biofilm development	119.6 ± 13.2
Mutants with decreased cytotoxicity				
PA14_05320	PA0408	*pilG*	Type IV pilus response regulator; required for pilus extension and twitching motility	28.8 ± 4.4
PA14_05330	PA0409	*pilH*	Type IV pilus response regulator; required for pilus retraction	25.8 ± 1.5
PA14_50220	PA1097	*fleQ*	Flagella major transcriptional regulator; cyclic-di-GMP responsive; potential FleSR modulator	77.6 ± 12.7
PA14_50200	PA1098	*fleS*	Two-component sensor kinase; required for hook and basal body protein biosynthesis for flagellum assembly	20.6 ± 6.7^‡^
PA14_50180	PA1099	*fleR*	Two-component response regulator; required for hook and basal body protein biosynthesis for flagellum assembly	12.3 ± 0.8^‡^
PA14_16500	PA3702	*wspR*	Two-component response regulator with GGDEF domain; c-di-GMP regulation	16.8 ± 3.7
PA14_12780	PA3948	*rocA1*	Two-component response regulator; cyclic-di-GMP regulation	53.2 ± 0.9
PA14_60260	PA4547	*pilR*	Two-component response regulator; required for pilus expression and therefore for type IV pilus biosynthesis	14.6 ± 4.5
PA14_68230	PA5165	*dctB*	Two-component sensor kinase; regulates a C4-dicarboxylate transport system with DctD	14.9 ± 3.5
PA14_69470	PA5261	*algR*	Alginate biosynthesis regulatory protein	33.2 ± 11.7
No major change in cytotoxicity				
PA14_00430	PA0034		Probable two-component response regulator	108.9 ± 5.1
PA14_01860	PA0150		Probable transmembrane sensor kinase	101.4 ± 3.0
PA14_02250	PA0178	*cheA*	Probable two-component sensor kinase	107.5 ± 4.1
PA14_02260	PA0179		Probable two-component response regulator	103.1 ± 4.1
PA14_06060	PA0463	*creB*	Two-component response regulator; catabolism and motility	94.2 ± 3.3
PA14_06070	PA0464	*creC*	Two-component sensor kinase; catabolism and motility	109.9 ± 5.4
PA14_07820	PA0600	*agtS*	Two component ensor kinase; amine uptake	95.9 ± 7.0
PA14_54510	PA0756		Probable two-component response regulator	103.3 ± 1.3
PA14_54500	PA0757		Probable two-component sensor kinase	109.4 ± 4.9
PA14_52240	PA0930	*pirS*	Two-component sensor kinase; iron acquisition	109.9 ± 3.5
PA14_49440	PA1157		Probable two-component response regulator	107.5 ± 3.8
PA14_49420	PA1158		Probable two-component sensor kinase	113.8 ± 5.7
PA14_49180	PA1179	*phoP*	Two-component response regulator; regulates cytotoxicity and resistance to cationic peptides	104.4 ± 1.8
PA14_49160	PA1243		Probable sensor kinase /regulator hybrid	94.0 ± 8.5
PA14_47390	PA1301		Probable transmembrane sensor kinase	111.2 ± 8.2
PA14_46980	PA1336	*aauS*	Two-component sensor kinase; amino acid uptake	110.1 ± 0.3
PA14_46850	PA1347		Probable transcriptional regulator	106.6 ± 1.3
PA14_46370	PA1396		Probably two-component sensor kinase	98.5 ± 3.5
PA14_46060	PA1422	*gbuR*	Transcriptional regulator	95.5 ± 4.5
PA14_45880	PA1437		Probable two-component response regulator	106.7 ± 3.1
PA14_45870	PA1438		Probable two-component sensor kinase	111.6 ± 4.5
PA14_43350	PA1636	*kpdD*	Two-component sensor kinase; potassium transport regulation	109.8 ± 4.5
PA14_42220	PA1727	*mucR*	Two component sensor kinase; alginate regulation	113.0 ± 4.5
PA14_41490	PA1785	*nasT*	Response regulator; nitrate/nitrite assimilation	103.2 ± 2.6
PA14_41270	PA1798	*parS*	Two-component sensor kinase; resistance to cationic peptides	93.9 ± 8.5
PA14_41260	PA1799	*parR*	Probable two-component response regulator; resistance to cationic peptides	107.7 ± 4.3
PA14_40570	PA1851		Probable two-component response regulator	104.4 ± 1.9
PA14_38970	PA1976	*ercS'*	Probable two-component sensor kinase; ethanol oxidation	112.7 ± 5.1
PA14_38900	PA1980	*eraR*	Probable two-component response regulator; ethanol oxidation	98.7 ± 5.0
PA14_37980	PA2051		Probable transmembrane sensor kinase	103.9 ± 5.7
PA14_36420	PA2177		Probable histidine sensor kinase	100.6 ± 4.6
PA14_32580	PA2479		Probable two-component response regulator	107.3 ± 4.7
PA14_31960	PA2523	*czcR*	Two-component response regulator; metal and imipenem resistance	107.4 ± 4.1
PA14_31950	PA2524	*czcS*	Two-component sensor kinase; metal and imipenem resistance	107.5 ± 1.0
PA14_30830	PA2572		Probable two-component response regulator	111.9 ± 5.2
PA14_30700	PA2583		Probable sensor kinase/response regulator hybrid	100.3 ± 6.5
PA14_29740	PA2656		Probable two-component sensor kinase	101.6 ± 6.5
PA14_29360	PA2687	*pfeS*	Two-component sensor kinase; iron acquisition	88.0 ± 4.8
PA14_27940	PA2798		Probable two-component response regulator	108.6 ± 3.4
PA14_27810	PA2809	*copR*	Two-component response regulator; copper resistance	106.4 ± 5.3
PA14_27800	PA2810	*copS*	Two-component sensor kinase; copper resistance	93.7 ± 2.3
PA14_27550	PA2824	*sagS*	Two-component sensor/regulator hybrid; motile/sessile switch, activates Gac/Hpt/Rsm system	88.9 ± 8.6
PA14_24720	PA3044	*rocS2*	Two-component sensor kinase; c-di-GMP regulation	96.9 ± 4.5
PA14_24710	PA3045	*rocA2*	Two-component response regulator; c-di-GMP regulation	103.2 ± 4.0
PA14_24350	PA3077	*cprR*	Probable two-component system regulatory protein; cationic peptide resistance	112.8 ± 9.5
PA14_24340	PA3078	*cprS*	Probable two-component sensor kinase; cationic peptide resistance	98.4 ± 6.2
PA14_22960	PA3191	*gtrS*	Probable two-component sensor kinase; virulence	107.3 ± 4.4
PA14_22940	PA3192	*gltR*	Two-component response regulator; virulence	98.6 ± 2.6
PA14_22730	PA3206		Probable two-component sensor kinase	102.5 ± 6.3
PA14_21700	PA3271		Probable two-component sensor kinase	105.9 ± 11.3
PA14_20820	PA3343		Probable two-component response regulator	108.8 ± 7.1
PA14_20780	PA3346		Probable two-component response regulator	105.7 ± 3.9
PA14_20000	PA3409	*hasS*	Probable Fe^2+^-dicitrate sensor kinase; diguanylate cyclase	102.5 ± 5.7
PA14_17670	PA3604	*erdR*	Two-component response regulator; ethanol oxidation	99.4 ± 10.1
PA14_16350	PA3714		Probable two-component response regulator	110.9 ± 5.3
PA14_13740	PA3878	*narX*	Two-component sensor kinase; nitrate respiration, biofilm, motility	100.5 ± 10.9
PA14_12820	PA3900	*fecR*	Probable transmembrane sensor protein	110.9 ± 5.6
PA14_12820	PA3946	*rocS1*	Two-component sensor kinase; c-di-GMP regulation	103.4 ± 7.4
PA14_12810	PA3947	*rocR*	Antagonist of rocA1 response regulator; c-di-GMP regulation with EAL domain	93.5 ± 1.9
PA14_11680	PA4032		Probable two-component response regulator	106.0 ± 2.6
PA14_11630	PA4036		Probable two-component sensor kinase	110.9 ± 3.8
PA14_10770	PA4112		Probable sensor/regulator hybrid	97.4 ± 9.4
PA14_09690	PA4196	*bfiR*	Probable two-component response regulator; biofilm formation	90.8 ± 9.9
PA14_09680	PA4197	*bfiS*	Two-component sensor kinase; biofilm formation	97.5 ± 2.9
PA14_55780	PA4293	*pprA*	Two-component sensor kinase; outer membrane permeability and resistance	108.8 ± 4.1
PA14_55810	PA4296	*pprB*	Probable two-component response regulator; outer membrane permeability and resistance	100.5 ± 6.8
PA14_56950	PA4381	*colR*	Probable DNA-binding response regulator; polymyxin resistance	103.8 ± 2.5
PA14_57140	PA4396		Probable two-component response regulator	103.0 ± 10.0
PA14_57170	PA4398		Probable two-component response regulator	105.5 ± 12.7
PA14_58300	PA4493	*roxR*	Two-component response regulator; regulates production of cyanide insensitive oxidase	107.0 ± 3.8
PA14_58320	PA4494	*roxS*	Two-component sensor kinase; regulates production of cyanide insensitive oxidase	108.1 ± 6.4
PA14_60250	PA4546	*pilS*	Two-component sensor kinase; type IV pili production	113.5 ± 5.3
PA14_63150	PA4776	*pmrA*	Two-component response regulator; cationic peptide resistance	104.3 ± 2.3
PA14_63160	PA4777	*pmrB*	Two-component sensor kinase; cationic peptide resistance	92.0 ± 10.8
PA14_64050	PA4843	*gcbA*	Probable two-component response regulator; diguanylate cyclase	108.8 ± 3.0
PA14_64880	PA4983		Probable two-component response regulator	108.5 ± 5.4
PA14_68250	PA5166	*dctD*	Two-component response regulator; C4-dicarboxylate metabolism	97.1 ± 3.6
PA14_68680	PA5199	*amgS*	Two-component sensor kinase; membrane stress, aminoglycoside resistance	91.5 ± 3.3
PA14_70750	PA5360	*phoB*	Two component response regulator; phosphate regulation and quorum sensing	105.9 ± 3.5
PA14_70760	PA5361	*phoR*	Two-component sensor kinase; phosphate regulation and quorum sensing	108.8 ± 3.3
PA14_72380	PA5483	*algB*	Two-component response regulator; alginate production	96.1 ± 3.2
PA14_72390	PA5484	*kinB*	Two-component sensor kinase; alginate production	93.6 ± 5.6
PA14_59770		*rcsB*	Two component response regulator; cup fimbria and biofilm formation	104.0 ± 5.2
PA14_59790		*pvrR*	Two component response regulator; EAL domain, c-di-GMP regulation, cup fimbria and biofilm	103.6 ± 4.7
PA14_59800		*pvrS*	Two component sensor kinase; c-di-GMP regulation, cup fimbria and biofilm	110.0 ± 4.2

^†^Mutants are listed in order of the PAO1 homolog. Mutants not in the screen included PA1637 *kpdE*—response regulator with KpdD, potassium transport; PA1786 *nasS*—sensor with *nasT*, nitrate/nitrite assimilation; PA2686 *pfeR*—response regulator with *pfeS*, iron acquisition; PA0929 *pirR*—response regulator with *pirS*, iron acquisition; PA3345 *htpB*—response regulator with *sagS*, activates Gac/Htp/Rsm system; PA3879 *narL*—response regulator with *narX*, nitrate respiration, biofilm, motility; PA3974 *ladS*—sensor, virulence, biofilm formation, cytotoxicity; PA4380 *colS*—sensor with *colR*, polymyxin resistance; PA4101 *bfmR*—response regulator with *bfmS*, biofilm development; PA4102 *bfmS*—sensor with *bfmR*, biofilm development; PA4856 *retS*—sensor, virulence, biofilm formation, cytotoxicity; PA4959 *fimX*—sensor (orphan?); contains GGDEF and EAL domains; cytotoxicity; PA5200 *amgR*—response regulator with *amgS*, membrane stress and aminoglycoside resistance; PA5262 *fimS*—sensor with *algR*, virulence, alginate biosynthesis, motility, biofilm; PA5511 *mifR*—response regulator with *mifS*, biofilm development; PA14_59780 *rcsC*—sensor with *rcsB*, biofilm formation. ^‡^The phenotype for these mutants was different between PA14 and PAO1 as described in the text.

## References

[1] Gellatly SL, Hancock REW (2013). *Pseudomonas aeruginosa*: new insights into pathogenesis and host defenses. Pathog Dis.

[2] Schurek KN, Breidenstein EBM, Hancock REW, Dougherty TJ, Pucci MJ (2012). *Pseudomonas aeruginosa*: a persistent pathogen in cystic fibrosis and hospital-associated infections. Antibiotic drug discovery and Development.

[3] Roy-Burman A, Savel RH, Racine S (2001). Type III protein secretion is associated with death in lower respiratory and systemic *Pseudomonas aeruginosa* infections. J Infect Dis.

[4] Hauser AR, Cobb E, Bodi M (2002). Type III protein secretion is associated with poor clinical outcomes in patients with ventilator-associated pneumonia caused by *Pseudomonas aeruginosa*. Crit Care Med.

[5] Mikkelsen H, McMullan R, Filloux A (2011). The *Pseudomonas aeruginosa* reference strain PA14 displays increased virulence due to a mutation in *ladS*. PLoS One.

[6] Ramirez JC, Fleiszig SM, Sullivan AB (2012). Traversal of multilayered corneal epithelia by cytotoxic *Pseudomonas aeruginosa* requires the phospholipase domain of ExoU. Invest Ophthalmol Vis Sci.

[7] Gooderham WJ, Hancock REW (2009). Regulation of virulence and antibiotic resistance by two-component regulatory systems in *Pseudomonas aeruginosa*. FEMS Microbiol Rev.

[8] Yeung AT, Bains M, Hancock RE (2011). The sensor kinase CbrA is a global regulator that modulates metabolism, virulence, and antibiotic resistance in *Pseudomonas aeruginosa*. J Bacteriol.

[9] Gooderham WJ, Gellatly SL, Sanschagrin F (2009). The sensor kinase PhoQ mediates virulence in *Pseudomonas aeruginosa*. Microbiology.

[10] Liberati NT, Urbach JM, Miyata S (2006). An ordered, nonredundant library of *Pseudomonas aeruginosa* strain PA14 transposon insertion mutants. Proc Natl Acad Sci USA.

[11] Lewenza S, Falsafi RK, Winsor G (2005). Construction of a mini-Tn5-luxCDABE mutant library in *Pseudomonas aeruginosa* PAO1: A tool for identifying differentially regulated genes. Genome Res.

[12] Jacobs MA, Alwood A, Thaipisuttikul I (2003). Comprehensive transposon mutant library of *Pseudomonas aeruginosa*. Proc Natl Acad Sci USA.

[13] Macfarlane ELA, Kwasnicka A, Ochs MM (1999). PhoP-PhoQ homologues in *Pseudomonas aeruginosa* regulate expression of the outer-membrane protein OprH and polymyxin B resistance. Mol Microbiol.

[14] Breidenstein EB, Khaira BK, Wiegand I (2008). Complex ciprofloxacin resistome revealed by screening a *Pseudomonas aeruginosa* mutant library for altered susceptibility. Antimicrob Agents.

[15] Choi KH, Gaynor JB, White KG (2005). A Tn7-based broad-range bacterial cloning and expression system. Nat Methods.

[16] Choi KH, Schweizer HP (2006). Mini-Tn7 insertion in bacteria with single attTn7 sites: example *Pseudomonas aeruginosa*. Nat Protoc.

[17] Gruenert DC, Basbaum CB, Welsh MJ (1988). Characterization of human tracheal epithelial cells transformed by an origin-defective simian virus 40. Proc Natl Acad Sci USA.

[18] Yeung AT, Torfs EC, Jamshidi F (2009). Swarming of *Pseudomonas aeruginosa* is controlled by a broad spectrum of transcriptional regulators, including MetR. J Bacteriol.

[19] Overhage J, Lewenza S, Marr AK (2007). Identification of genes involved in swarming motility using a *Pseudomonas aeruginosa* PAO1 mini-Tn5-*lux* mutant library. J Bacteriol.

[20] O'Toole GA, Kolter R (1998). Flagellar and twitching motility are necessary for *Pseudomonas aeruginosa* biofilm development. Mol Microbiol.

[21] Gellatly SL, Needham B, Madera L (2012). The *Pseudomonas aeruginosa* PhoP-PhoQ two-component regulatory system is induced upon interaction with epithelial cells and controls cytotoxicity and inflammation. Infect Immun.

[22] Wu W, Badrane H, Arora S (2004). MucA-mediated coordination of type III secretion and alginate synthesis in *Pseudomonas aeruginosa*. J Bacteriol.

[23] Intile PJ, Diaz MR, Urbanowski ML (2014). The AlgZR two-component system recalibrates the RsmAYZ posttranscriptional regulatory system to inhibit expression of the *Pseudomonas aeruginosa* type III secretion system. J Bacteriol.

[24] Carterson AJ, Morici LA, Jackson DW (2004). The transcriptional regulator AlgR controls cyanide production in *Pseudomonas aeruginosa*. J Bacteriol.

[25] Whitchurch CB, Alm RA, Mattick JS (1996). The alginate regulator AlgR and an associated sensor FimS are required for twitching motility in *Pseudomonas aeruginosa*. Proc Natl Acad Sci USA.

[26] Lizewski SE, Lundberg DS, Schurr MJ (2002). The transcriptional regulator AlgR is essential for *Pseudomonas aeruginosa* pathogenesis. Infect Immun.

[27] Jiang J, Gu BH, Albright LM (1989). Conservation between coding and regulatory elements of *Rhizobium meliloti* and *Rhizobium leguminosarum*
*dct* genes. J Bacteriol.

[28] Wang YP, Birkenhead K, Boesten B (1989). Genetic analysis and regulation of the *Rhizobium meliloti* genes controlling C4-dicarboxylic acid transport. Gene.

[29] Valentini M, Storelli N, Lapouge K (2011). Identification of C4-dicarboxylate transport systems in *Pseudomonas aerguinosa* PAO1. J Bacteriol.

[30] Ritchings BW, Almira EC, Lory S (1995). Cloning and phenotypic characterization of *fleS* and *fleR*, new response regulators of *Pseudomonas aeruginosa* which regulate motility and adhesion to mucin. Infect Immun.

[31] Duan Q, Zhou M, Zhu L (2013). Flagella and bacterial pathogenicity. J Basic Microb.

[32] Haiko J, Westerlund-Wikström B (2013). The role of the bacterial flagellum in adhesion and virulence. Biology.

[33] Kohler T, Curty LK, Barja F (2000). Swarming of *Pseudomonas aeruginosa* is dependent on cell-to-cell signaling and requires flagella and pili. J Bacteriol.

[34] Dasgupta N, Wolfgang MC, Goodman AL (2003). A four-tiered transcriptional regulatory circuit controls flagellar biogenesis in *Pseudomonas aeruginosa*. Mol Microbiol.

[35] Arora SK, Ritchings BW, Almira EC (1997). A transcriptional activator, FleQ, regulates mucin adhesion and flagellar gene expression in *Pseudomonas aeruginosa* in a cascade manner. J Bacteriol.

[36] Mattick JS (2002). Type IV pili and twitching motility. Annu Rev Microbiol.

[37] Kelly NM, Kluftinger JL, Pasloske BL (1989). *Pseudomonas aeruginosa* pili as ligands for nonopsonic phagocytosis by fibronectin-stimulated macrophages. Infect Immun.

[38] Chiang P, Burrows LL (2003). Biofilm formation by hyperpiliated mutants of *Pseudomonas aeruginosa*. J Bacteriol.

[39] Vallet I, Olson JW, Lory S (2001). The chaperone/usher pathways of *Pseudomonas aeruginosa*: identification of fimbrial gene clusters (*cup*) and their involvement in biofilm formation. Proc Natl Acad Sci USA.

[40] Harmsen M, Yang L, Pamp SJ (2010). An update on *Pseudomonas aeruginosa* biofilm formation, tolerance, and dispersal. FEMS Immunol Med Microbiol.

[41] Craig L, Pique ME, Tainer JA (2004). Type IV pilus structure and bacterial pathogenicity. Nat Rev Microbiol.

[42] D'Argenio DA, Calfee MW, Rainey PB (2002). Autolysis and autoaggregation in *Pseudomonas aeruginosa* colony morphology mutants. J Bacteriol.

[43] Jenal U, Malone J (2006). Mechanisms of cyclic-di-GMP signaling in bacteria. Annu Rev Genet.

[44] Giraud C, Bernard CS, Calderon V (2011). The PprA-PprB two-component system activates CupE, the first non-archetypal *Pseudomonas aeruginosa* chaperone-usher pathway system assembling fimbriae. Environ Microbiol.

[45] Sato A, Iwasaki A (2005). Peyer's patch dendritic cells as regulators of mucosal adaptive immunity. Cell Mol Life Sci.

[46] Wu X, Wang H, Zhao X (2008). Antimicrobial studies with the Pseudomonas aeruginosa two-allele library require caution. Antimicrob Agents.

[47] Held K, Ramage E, Jacobs M (2012). Sequence-verified two-allele transposon mutant library for *Pseudomonas aeruginosa* PAO1. J Bacteriol.

[48] Macfarlane EL, Kwasnicka A, Hancock RE (2000). Role of *Pseudomonas aeruginosa* PhoP-PhoQ in resistance to antimicrobial cationic peptides and aminoglycosides. Microbiology.

[49] Yeung AT, Janot L, Pena OM (2014). Requirement of the *Pseudomonas aeruginosa* CbrA sensor kinase for full virulence in a murine acute lung infection model. Infect Immun.

